# The impact of ventricular morphology on wall stress and ventricular strain in Fontan patients

**DOI:** 10.1186/1532-429X-18-S1-O30

**Published:** 2016-01-27

**Authors:** Sunil J Ghelani, Steven D Colan, David M Harrild, Andrew J Powell, Tal Geva, Rahul H Rathod

**Affiliations:** Cardiology, Boston Children's Hospital, Boston, MA USA

## Background

Adverse outcomes are increasingly common in patients after the Fontan operation as they approach adulthood. Right ventricular (RV) morphology, ventricular dilation, and diminished ventricular strain have all been independently associated with death or heart transplant. The aim of this study was to assess the impact of ventricular morphology on ventricular stress/strain relationship in patients late after the Fontan operation using cardiac magnetic resonance (CMR).

## Methods

Clinical CMR studies in patients with Fontan circulation between January 1, 2005 and June 30, 2013 were retrospectively analyzed. Ventricular mass and volumes were calculated using commercially available software (QMass, Medis Medical Imaging Systems, Leiden, the Netherlands). Global circumferential strain (GCS) was measured for the single or dominant ventricle at the mid ventricular level using commercial software (2D CPA MR, TomTec imaging systems, Unterschleissheim, Germany). End systolic wall stress (WS) was calculated for the ventricle using a thick-walled sphere model. Arm cuff mean arterial blood pressure was used as a surrogate for left ventricular end-systolic pressure.

## Results

**Figure 1:**
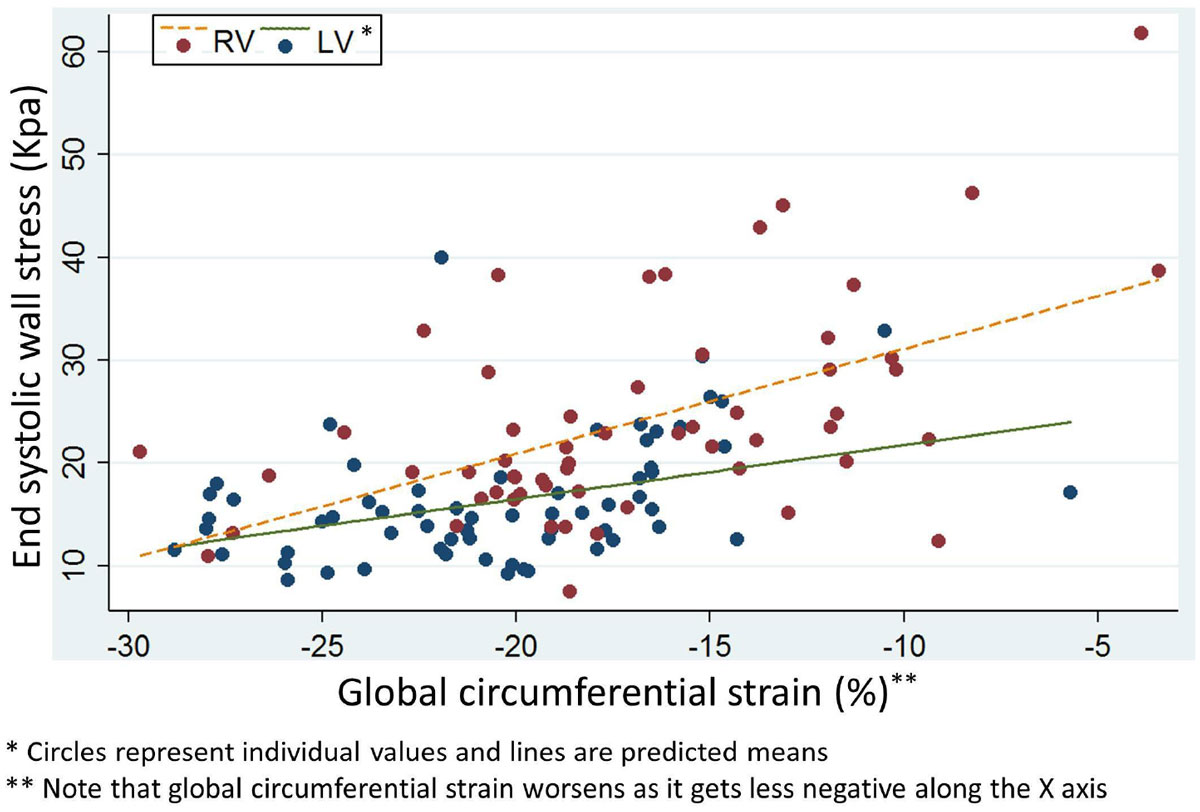
**Relationship between ventricular wall stress and circumferential strain in dominant left and right ventricles in the Fontan circulation**.

The study population consisted of 126 patients (64% male) with median age of 16.9 years (IQR 12.3-23.3) and a median age at Fontan surgery of 3.2 years (IQR 2.3-5). The dominant ventricular morphology was left (LV) in 52% and RV in 48%. Compared with LV dominant Fontan patients, RV-dominant patients were younger (16 ± 8 vs 21 ± 10 years; p = 0.002), had a higher heart rate (87 ± 20 vs. 77 ± 17 beats per minute; p = 0.006), and lower mean arm blood pressure (76 ± 12 vs. 81 ± 10 mmHg; p = 0.014). RV-dominant patients also had higher WS (24 ± 10 vs. 16 ± 6 KPa; p < 0.001) and lower GCS (-17 ± 5 vs. -20 ± 5%; p < 0.001). Multivariable linear regression analysis controlling for age, heart rate, and body surface area demonstrated that each unit reduction in GCS was associated with an increase in WS of 0.8 KPa (95% CI 0.53-1.1; p < 0.001), and RV morphology was associated with higher WS independent of GCS (p = 0.003). Relationships between WS and strain in the left and right ventricles are shown in Figure 1. There was a tendency for the WS-GCS relationship to be steeper for RVs (p = 0.08) consistent with increased load-dependence for RVs. Ventricular mass to end-systolic volume ratio was significantly lower for the RV (0.84 ± 0.5 gm/ml; p < 0.001) as compared with the LV (1.3 ± 0.5 gm/ml; p < 0.001), consistent with inadequate RV hypertrophy.

## Conclusions

Compared to Fontan patients with a functional single LV, those with a dominant RV have an adverse stress-strain relationship. Inadequate ventricular hypertrophy is the primary driver for higher WS in single RVs. This may, in part, explain the previously reported unfavorable outcomes in Fontan patients with RV morphology as compared to LV morphology.

